# The Central Region of Testican-2 Forms a Compact Core and Promotes Cell Migration

**DOI:** 10.3390/ijms21249413

**Published:** 2020-12-10

**Authors:** Anja Krajnc, Aljaž Gaber, Brigita Lenarčič, Miha Pavšič

**Affiliations:** 1 Department of Chemistry and Biochemistry, Faculty of Chemistry and Chemical Technology, University of Ljubljana, SI-1000 Ljubljana, Slovenia; anja.kers@fkkt.uni-lj.si (A.K.); aljaz.gaber@fkkt.uni-lj.si (A.G.); brigita.lenarcic@fkkt.uni-lj.si (B.L.); 2 Department of Biochemistry, Molecular and Structural Biology, Jožef Stefan Institute, SI-1000 Ljubljana, Slovenia

**Keywords:** testican, SPOCK, calcium-binding, structural model, cell migration

## Abstract

Testicans are modular proteoglycans of the extracellular matrix of various tissues where they contribute to matrix integrity and exert cellular effects like neurite outgrowth and cell migration. Using testican-2 as a representative member of the family, we tackle the complete lack of general structural information and structure–function relationship. First, we show using isothermal titration calorimetry and modeling that extracellular calcium-binding domain (EC) has only one active calcium-binding site, while the other potential site is inactive, and that testican-2 is within extracellular matrix always in the calcium-loaded form. Next, we demonstrate using various prediction methods that N- and C-terminal regions plus interdomain connections are flexible. We support this by small-angle X-ray-scattering analysis of C-terminally truncated testican-2, which indicates that the triplet follistatin-EC-thyroglobulin domain forms a moderately compact core while the unique N-terminal is disordered. Finally, using cell exclusion zone assay, we show that it is this domain triplet that is responsible for promoting cell migration and not the N- and C-terminal regions.

## 1. Introduction

Testicans/SPOCKs are proteoglycans of the vertebrate extracellular matrix (ECM). Together with other proteoglycans and glycoproteins they form a complex network which provides hydration, compression resistance, and binding sites for soluble and cell surface molecules, thereby extending the functionality of the fibrous components of the ECM (collagens, fibronectins and elastans) [1,2]. All these interconnected components are critically involved in intercellular communication and migration, which are both hallmarks of normal physiological processes like morphogenesis and differentiation, as well as pathological conditions, for example, cancer cell invasion [3]. Recently, a member of the testican family, testican-1, has been shown to be involved in the proliferation, migration and invasion of cancer cells via the PI3K/Akt and Wnt/β-catenin pathway, and therefore represents a potential therapeutical target [4,5]. However, despite their involvement in these and other important processes outlined below, the data on their structure and structure–function relationship are virtually non-existent.

**Figure 1:**
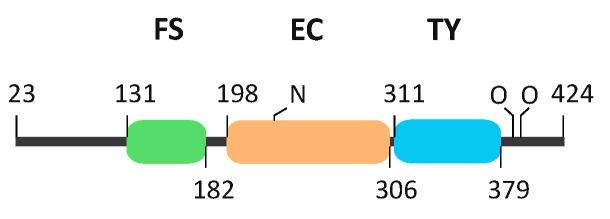
Schematic representation of testican-2 domain architecture. Full-length testican-2 is composed of N- and C-terminal regions surrounding the three central domains: follistatin (FS, green), calcium-binding (EC, orange) and thyroglobulin (TY, blue). Labels N and O mark N- and O-glycosylation sites, respectively. The scheme depicts mature testican-2 without signal peptide, numbering refers to interdomain boundaries.

The testican protein family has three members—the paralogues testican-1, -2 and -3. The multiple sequence alignment reveals a 34% amino acid sequence identity (pairwise alignments 42–51% identity). They share a common modular organization (Figure 1)—a unique N-terminal region followed by a follistatin-like domain (FS; containing a Kazal module), calcium-binding domain (EC), thyroglobulin type-1 domain (TY), and a C-terminal tail with heparan/chondroitin sulfate glycosaminoglycan (GAG) attachment sites at two serine residues [6,7,8]. Testicans are also termed SPOCKs for their SPARC/osteonectin (FS–EC), CWCV (TY) and Kazal-like domains, and the presence of an FS–EC pair classifies them as SPARC/BM-40/osteonectin family members together with SPARC, Hevin, SMOCs and FstI-1 [9]. Of these, BM-40 is the only member with a determined structure of the central FS–EC domain pair [10].

All three testicans are expressed in different tissues throughout the human body, with the highest levels of expression in the brain [7,8,11]. Their function appears to be partially redundant, since testican-1 knock-down mice showed no pronounced morphological or behavioral abnormalities, were fertile and had normal lifespans [12]. Likewise, testican-3-deficient mice were viable, fertile and did not show obviously abnormal phenotype [11], however, at specific stages of development, the lack of testican-3 resulted in brain structural abnormalities [13]. Similarly, not the lack but the presence of an abnormal protein can have serious consequences—a missense mutation D80V of testican-1 manifests itself as disruptions of major neuronal structures and developmental delay [14]. In addition to this, several specific roles have been attributed to various testicans. Testican-1 inhibits the activity of cathepsin L in vitro via its TY domain [15], and both testican-1 and -3 inhibit the activity of membrane-type proteinases (MT1-MMP and MT2-MMP) in astrocytic tumors [16], although recent research indicates that MMP inhibition is not direct [17]. Testican-2 was reported to counter the inhibitory action of testican-1 and -3 and supports MT-MMP-mediated cell migration [18]. Testicans evidently participate in a diverse set of normal and pathological cellular processes, including cell attachment and migration [5,19], neurite growth [20], epithelial-to-mesenchymal transition and cancer progression [21,22,23,24], visual processing [25], and Alzheimer disease [26]. The functions are largely attributed to their protein part. In most of the above reports, the mechanism of action was not described in detail at the molecular level, also due to the lack of structural data.

To gain insight into the spatial organization of testican domains and to connect this to the protein function, we focused on testican-2 as a representative member of the family. We used a recombinant protein lacking attached GAGs (achieved by expression in insect cells and C-terminal truncation), therefore the discovered properties are attributed solely to the protein part of the testican-2. We characterized its calcium-binding properties in terms of affinity and demonstrated that calcium binding has a positive effect on overall structure stabilization. By using small-angle X-ray-scattering complemented by structural modeling, we show that the central FS–EC–TY domain triplet forms a moderately compact structural core. Finally, we demonstrate that it is this central domain triplet that promotes cell migration in both testican-1 and -2.

## 2. Results

### 2.1. EC Domain of Testican-2 Binds a Single Calcium Ion

To determine stoichiometry and affinity of calcium binding, we performed detailed sequence and structural model analysis and supported it with data from ITC measurements of pure recombinant testican-2.

**Figure 2:**
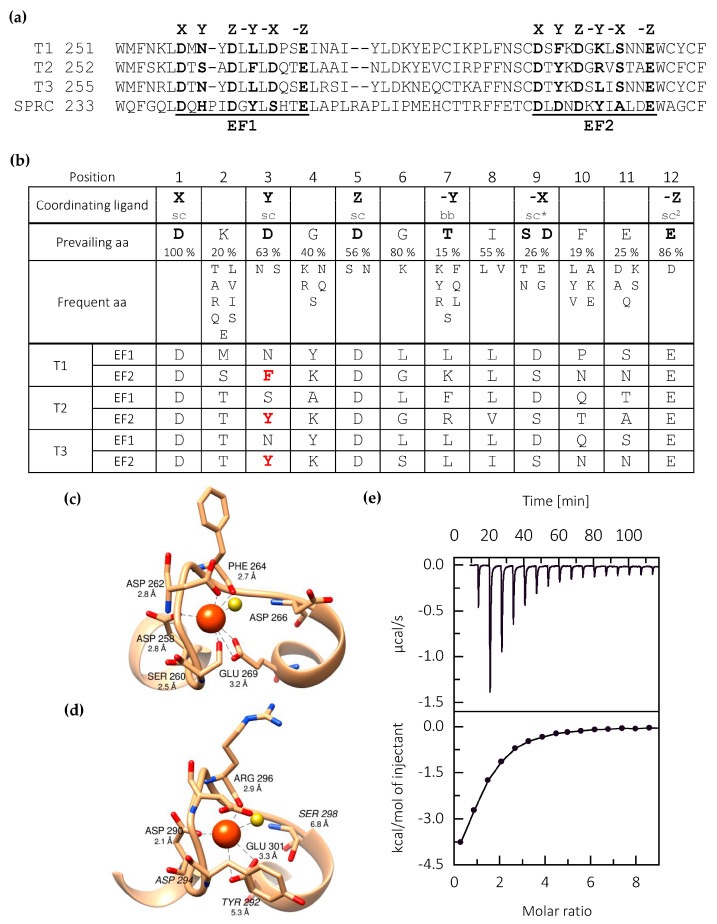
Calcium-binding EF-hand motifs in testicans. (**a**) Amino acid sequence alignment of both EF-hand motifs of the three testicans paralogues (T1, T2 and T3) and of the representative member of the SPARC protein family BM-40 (SPRC). Residues at sites critical for Ca^2+^ coordination are shown in bold. (**b**) Amino acid preferences of the EF-hand loop. The most common residues forming the Ca^2+^ coordination sphere and their loop positions are shown in bold. Coordination occurs either via side chain atoms (sc) or through the backbone atoms (bb). The asterisk marks the site where a water molecule serves as a bridge between the side chain and calcium ion. Sc2 indicates bidentate ligand. For all three testicans, residues in both EF1 and EF2 motif are listed; those significantly diverging from the EF-hand consensus sequence are shown in red. (**c**,**d**) Model of the EF1 (**c**) and EF2 (**d**) of testican-2. Residues forming the proposed coordination sphere are shown as sticks. Distance measurements between Ca^2+^ and potential coordinating atoms are represented by dashed lines. Calcium atom and water molecule are represented as orange and yellow spheres, respectively. (**e**) Titration (ITC) of C-terminally truncated testican-2 (T2ΔC) with calcium. The upper panel represents the raw heat of binding as a function of time, and the lower panel shows integrated heat change after subtracting heat of dilution at different molar ratios. Results indicate a stoichiometry of 1 Ca^2+^ ion per 1 protein molecule.

Sequence alignment and motif identification using ScanProsite [27] revealed that all three testicans have two potential calcium-binding sites, corresponding to two EF-hand motifs within the EC domain (Figure 2a). Such a motif, composed of two short α-helices linked by a loop region, is generally known as a calcium-binding motif and is present in various proteins. Next, we performed detailed analysis of the two motifs at critical sites (labeled X, Y, Z, -Y, -X and -Z) (Figure 2b) according to the Prosite pattern ID PS00018 [28]. The EF1 motif of all three testicans is in complete agreement with the consensus sequence and would, in theory, allow Ca^2+^ binding. In SPARC, where both EF-hand motifs bind Ca^2+^, the EF1 motif slightly deviates from the canonical sequence—the Y site is occupied by a non-canonical His residue, which forms a *cis*-peptide bond with the subsequent Pro residue (insertion with regard to consensus sequence), thus enabling coordination of Ca^2+^ by carbonyl oxygen of the Pro residue [10]. On the contrary, the EF2 motif of all three testicans significantly deviates from the consensus and SPARC EF2 motif sequence at position Y. Here, Phe (testican-1) or Tyr (testican-2 and -3) occupy the site, which is in a consensus motif preferentially occupied by a small polar residue (Asp, Asn, Ser). The sequence analysis therefore indicates that, in testicans, only the EF1 motif is able to bind a calcium ion.

Further insight into the Ca^2+^-binding properties of testican-2 is provided by the analysis of the structural models of its two EF-hand regions (Figure 2c,d). The coordination sphere of the EF1 is expectedly formed by the carboxyl side chain atoms of Asp 258 (position X), Asp 262 (position Z) and Glu 269 (position -Z), water molecule bridge to Asp266 (position -X), side chain of Ser 260 (position Y), and backbone of Phe 264 (position -Y). The distances between the Ca^2+^ ion and the coordinating atoms are in the range 2.8–3.8 Å, thus allowing successful coordination. However, in the case of EF2, the distances between Ca^2+^ ion and side chain oxygen atoms of Tyr 292 (position Y) and Asp 294 (position Z), with values of 7.8 and 5.8 Å, respectively, exceed the limit that would allow successful coordination considering the detailed analysis of EF motifs from other proteins [29,30]. Theoretically, it could be possible that coordination at Y and Z positions is mediated via Ca^2+^-proximal backbone atoms and not via Ca^2+^-distal side chain atoms, however, this would require considerable changes in the torsion angles of the cognate residues, thereby introducing significant structural perturbations to the EF2-hand motif as a whole. On the basis of the presented model, the coordination sphere of EF2 is therefore expected to be incomplete and successful Ca^2+^ coordination is unlikely, thus supporting the conclusion based on sequence analysis.

Next, calcium-binding affinity and stoichiometry were analyzed using isothermal titration calorimetry. Thermogram (Figure 2e) exhibits a single exothermic event at a molar ratio of n = 1.3 ± 0.2, corresponding to binding of a single calcium ion with a *K*_d_ = 17.1 ± 3.4 µM. Other thermodynamic parameters are listed in Table 1. Therefore, ITC measurements are in line with sequence and structural model analysis indicating a single Ca^2+^ binding site located within the EF1-hand motif.

### 2.2. Calcium Binding Stabilizes the Central Core of Testican-2

The polypeptide chain regions corresponding to the FS, EC and TY domains are, on the basis of homology to known structures, expected to fold into structurally well-defined units. However, N- and C-terminal regions are unique and unknown from a structural point of view.

**Figure 3:**
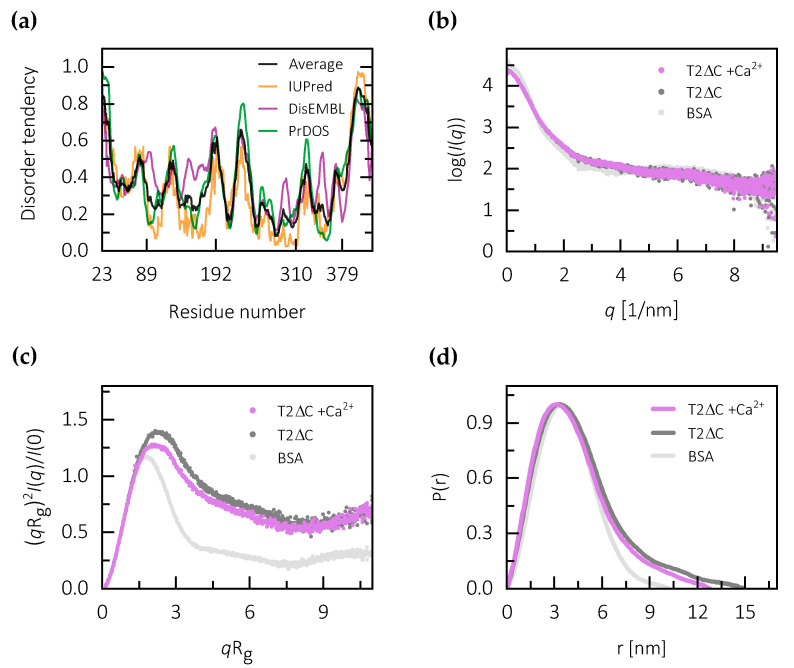
Disorder analysis of testican-2. (**a**) Disorder tendency plot for testican-2. The average value from all three tools used is represented in black. Marked residue numbers correspond to approximate borders between N-region, FS, EC and TY domains, and the C-region. (**b**) Scattering profile *I*(*q*) *vs. q* as log-linear plots for calcium-free (T2ΔC), calcium-loaded testican (T2ΔC + Ca^2+^) and BSA (used as a standard). (**c**) Dimensionless Kratky plots showing bell-shaped peaks with elevated plateau values at high *q*, indicating that the conformation of T2ΔC is not as compact as of BSA, for which a defined Gaussian-like peak was observed. (**d**) Normalized bell-shaped P(r) versus r profiles with D_max_ of 13.8 nm for a calcium-loaded sample and 14.5 nm for a calcium-free sample. The tail-like extension of the curve at higher r values for testican as compared to BSA indicates flexibility.

To obtain insight into the potential of N- and C-terminal regions of testicans to form compact structural units, we first performed basic sequence analysis. The N-terminal region shows no significant similarity to proteins of known structure. There is also no significant similarity to proteins outside of the testican family, as revealed by BLAST search [31]. The sequence complexity of the C-terminal region located just after the TY domain is low and its striking feature is a high proportion of negatively charged residues, as revealed by compositional bias analysis using fLPS [32]. Within the C-terminal region of testican-2 there are 19 residues bearing a negative charge (Asp, Glu), as opposed to only 1 residue bearing a positive charge (Lys) out of a total of 45 residues. Such uniformly charged regions are often structurally disordered due to self-repulsion and, consequently, unable to fold into compact structures [33,34]. Further insight into the disorder tendency is provided by the results of disorder prediction using IUPred [35], DisEMBL [36] and PrDOS [37], which all indicate that both N- and C-terminal regions of testican-2 have higher-than-average tendency to be structurally disordered. The disorder tendency is especially pronounced in the C-terminal region (Figure 3a). Other peaks in the disorder tendency plot are in good correlation with regions linking the FS, EC and TY domains (Figure 1 and Figure 3). The results of analogous predictions for testican-1 and -3 are similar (Figure S3), thus indicating that the unstructured N- and C-terminal plus linking regions are a general feature of the testican family. 

Further insight into disorder tendency is provided by small-angle X-ray-scattering data collected using the C-terminally truncated testican-2 (T2ΔC) in both the presence and absence of calcium ions (Figure 3b). The full-length protein and N-, C-terminally truncated form (T2ΔNC) exhibited aggregation, which prevented detailed analysis of the data.

The normalized Kratky plot for T2ΔC indicates that testican-2 without the C-terminal region, in both the presence and absence of calcium, displays a considerable degree of flexibility as compared to BSA. T2ΔC has a less globular shape which is indicated by an up-curving in the region with high *qR*_g_ values, and the maximum shifted to higher *qR*_g_ values (Figure 3c). The calcium-free form exhibits a slightly higher degree of flexibility compared to the calcium-loaded form. Still, the overall high degree of structural flexibility in both forms could be attributed to the N-terminal region, which is predicted to be structurally disordered, plus the linking regions between domains.

The analysis of Guinier region of the scattering data gave a linear fit with the values of *R*_g_ of 3.41 nm for calcium-free form, and an *R*_g_ of 3.24 nm for the calcium-loaded form (Table S1). This indicates that calcium binding results in a more compact structure, with a decrease in *R*_g_ of approximately 5%. Likewise, maximum linear dimension (D_max_) determined from the pair-density distribution function (P(r)) (Figure 3d) differs in the same manner, with values of 14.0 and 12.6 nm for calcium-free and calcium-loaded forms, respectively. The P(r) functions for both forms exhibit a slightly longer tail compared to the compact globular protein represented by bovine serum albumin (BSA), possibly corresponding to the N-terminal region in a structurally disordered or extended state. Therefore, the molecular parameters indicate that calcium binding stabilizes the structure and results in a higher level of compactness of the T2ΔC core, while at least the N-terminal region remains flexible.

### 2.3. The FS–EC–TY Domain Triplet Forms a Compact Structural Core

**Figure 4:**
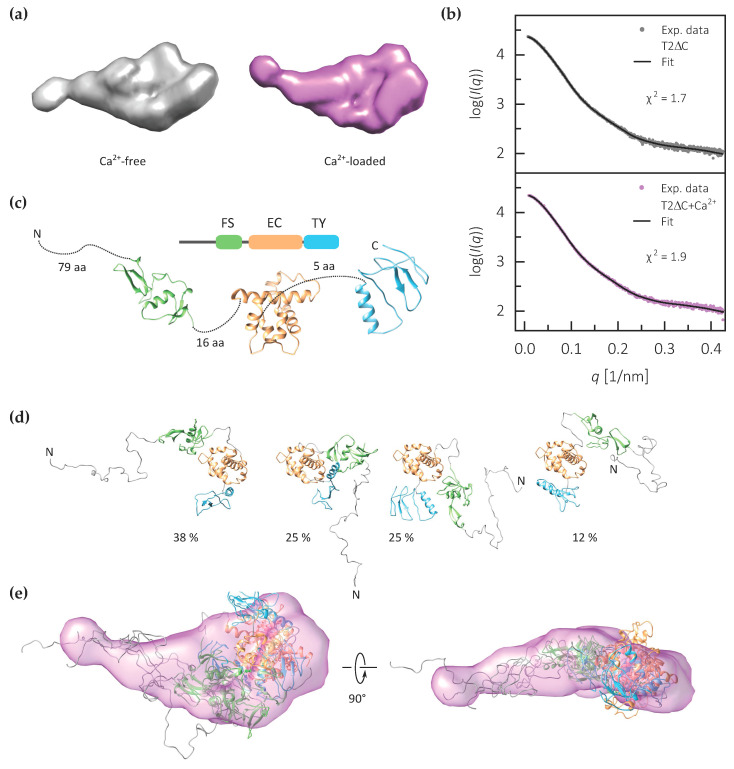
Structural models C-terminally truncated testican-2 based on SAXS data. (**a**) *Ab initio* molecular envelope of calcium-free and calcium-loaded T2ΔC generated using DAMMIF+DAMAVER with (**b**) corresponding fit to the experimental data (χ^2^). (**c**) Schematic representation of testican-2 structure with corresponding domain homology models. (**d**) Individual models of T2ΔC from the EOM-generated ensemble with their percentage contribution. The orientation of all four models is equivalent with regard to the EC domain. (**e**) Superposition of the four EOM-generated models with the averaged ab initio reconstructed molecular envelope of T2ΔC as shown in (**c**). Follistatin domain (FS) is shown in green, calcium-binding domain (EC) in orange, thyroglobulin domain (TY) in blue, and the N-terminal region plus the interdomain-linking regions in gray.

We used the collected structural data to build an integrative model of C-terminally truncated testican-2. As a first step, SAXS data were used to generate ab initio models of both calcium-free and calcium-loaded forms (Figure 4a). Models for both are in agreement with the experimental data (χ^2^ values of 1.7 and 1.9) and have a relatively compact core, most probably corresponding to the FS–EC–TY domain triplet, and an extension, most probably corresponding to the structurally disordered N-terminal region.

To prepare a more detailed model of the calcium-loaded testican-2, representing the physiologically relevant calcium-loaded form, we first generated separate homology models of the three central domains—FS, EC and TY (Figure 4b). Next, we employed EOM using the SAXS data and homology models (Figure 4c) to generate an ensemble of T2ΔC models bearing both the FS–EC–TY domain triplet as well as the structurally disordered N-terminal region. The final ensemble of four models fits the SAXS data, with an *χ*^2^ value of 1.8; the *R*_g_ and D_max_ values of the ensemble are 3.33 and 12.7 nm, respectively. Overall, the final selected models tend to be more compact than those in the random pool, which is shown by the shift to smaller *R*_g_ values of selected models compared to the distribution derived from the initial pool (Figure S7).

In three of the four models from the ensemble, the FS, EC and TY domains are arranged in a triangular fashion forming a more compact core, however, due to the low resolution of the method the relative orientation of the domains cannot be exactly defined (Figure 4d). The N-terminal region is modelled as disordered. Therefore, both the EOM-generated models and the ab initio model of the calcium-loaded form indicate that the three domains that are expected to be structurally ordered form a core of the T2ΔC (Figure 4e).

### 2.4. Protein Part of Testicans Promotes Cell Migration

The positive effect of testican-1 on cell migration has already been described [5]. To draw parallels with testican-2, its structural model and specific regions of its protein part, we evaluated the effect of exogenously added testican-1 and -2 constructs on the migration capacity of U373 cells using a cell exclusion zone assay.

**Figure 5:**
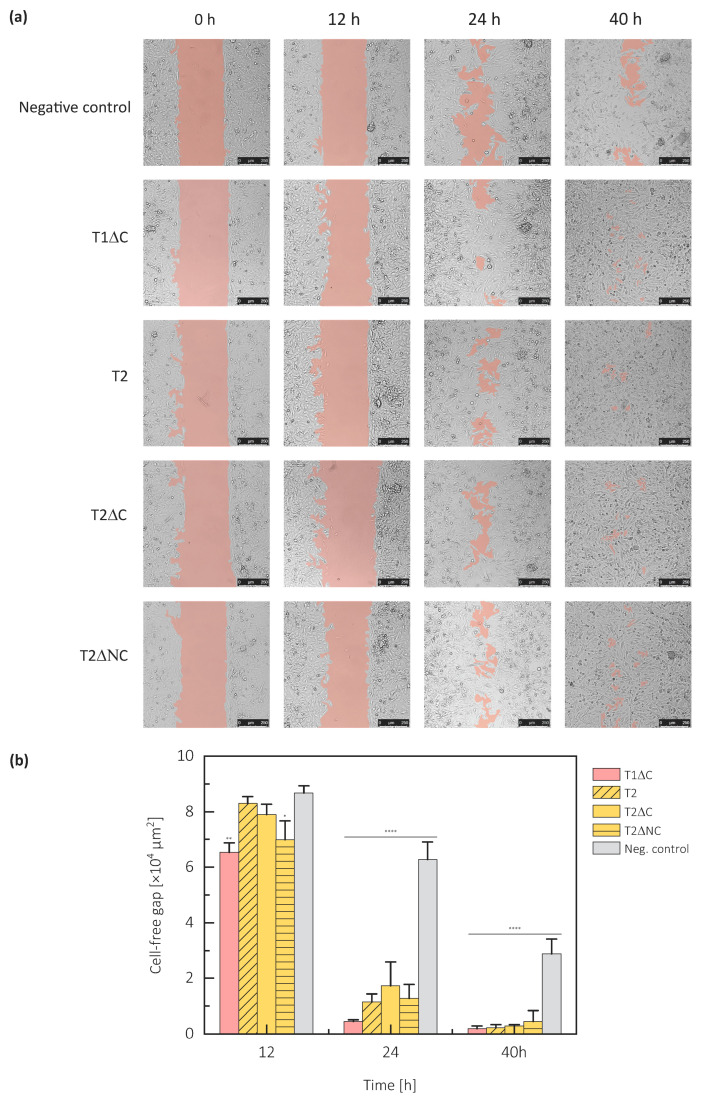
Cell exclusion zone assay reflecting the impact of extracellularly added testicans on cell migration. U373 cell monolayers with beforehand created gaps were incubated with 10 µg of different testican constructs and monitored with a confocal microscope. (**a**) Photographs taken at distinct time points (0, 12, 24, 40 h) after the addition of testican constructs (T1ΔC, T2, T2ΔC, and T2ΔNC) and BSA, showing faster gap closure in the presence of testicans regardless of the construct. The cell-free area is shown in salmon. (**b**) The quantified cell-free area in the presence of different testican constructs at different times, indicating there is a significant difference in cell migration in the presence of testicans, compared to the negative control (BSA). Values represent mean values of 3 independent measurements with s.d., * *p* < 0.0332, ** *p* < 0.0021, **** *p* < 0.0001, one-way ANOVA test with Tukey post hoc analysis.

We used full-length testican-2, C-terminally truncated testican-1 (T1ΔC) and -2 (T2ΔC), plus N- and C-terminally truncated testican-2 (T2ΔNC); all forms were without GAGs due to expression in insect cells, C-terminally truncated forms also had GAG attachment sites removed. Additionally, the apparent molecular mass in the range 37–50 kDa (Figure S2) for various constructs indicates the absence of GAGs. Compared to the negative control, all tested forms increased migration into the cell-free area. The gap was filled more than 20% faster in the presence of any form of the testican-1 or testican-2 (Figure 5).

A comparison of cell migration in the presence of full-length testican-2 and both of its truncated forms indicates that terminal regions are not required for its involvement in the cell migration process, since an approximately similar time was required for the gap closure (Figure 5). Therefore, it is the central domain triplet FS–EC–TY that is likely accountable for the cell migration enhancing function.

Testican-1 and testican-2 exhibited a comparable effect on the velocity of gap occupation, confirming once more the functional redundancy of the testican family members. To evaluate the cooperative action of different paralogues and their impact on cell migration, equimolar concentrations of different testican-2 constructs were mixed with T1ΔC. There were no obvious differences between cell migration in the presence of single or both testican-1 and -2 constructs, indicating that testicans do not act in an interdependent manner.

## 3. Discussion

For testicans, proteins of the vertebrate extracellular matrix with an especially high expression level in neuronal tissue, various roles have been ascribed, mainly linked to their effect on cell attachment and migration. Here, we provide basic structural data on testican-2, aiming to address the lack of available information on its structure and structure–function relationship. We believe that our findings are at least partially applicable to other two members of the testican family (testican-1 and -3), particularly in terms of their overall structural organization. First, testicans share the domain organization (Figure S1) where the central FS–EC–TY domain triplet is flanked by regions with higher disorder tendency. Next, the high sequence identity of 34% within these domains without any significant insertions/deletions (Figure S1), and conserved disulfide bridges within each of the domain, speak of structural similarity. The most significant differences in their amino acid sequences are within the C-terminal region, which are predicted to be disordered. Furthermore, their partial functional redundance could be attributed to their sequence similarity, and the specific functional aspects to their specific sequence features.

In their native forms, all three testicans are heavily O-glycosylated at the C-terminal region, where two GAG attachments sites are located [6,7,8]. In our study, we used recombinant human testican-1 and -2, which lack the GAG chains. This was achieved by truncation at the C-terminal end where these sites are located, and by using the insect cell expression system with limited capacity to produce such glycan structures [38]. It has already been demonstrated that human testican-2 produced in *S. frugiperda* cells does not bear extensive glycan moieties [39]. In addition to the full-length form, we prepared C- (T2ΔC) and N-, C-terminally truncated variants (T2ΔNC) to explore the structure of these regions and their effect on cell migration.

First, we addressed the calcium-binding characteristics of testican-2. By combining sequence analysis, structure modeling and experimental data obtained using isothermal titration calorimetry, we conclude that only the EF1-hand motif of testican-2 binds calcium with a *K*_d_ of 17.1 ± 3.4 µM, while EF2 motif is inactive. Calcium affinity of the same order of magnitude (*K*_d_ = 68 µM) has already been shown for testican-1 [40], however, the protein used in our study (T2ΔC) had regions on the N- and C-terminal side of the extracellular calcium-binding domain (EC), while the study on testican-1 was done using the isolated EC domain. Therefore, the difference in calcium affinity is not only due to the different protein itself, but could also be attributed to the presence/absence of flanking regions which, via a mutually stabilizing effect, affect thermodynamics of calcium binding, as was demonstrated for EC domain of BM-40 [10,41] and EF-motifs containing calmodulin-like domain of α-actinin-1 [42]. For instance, even though metal ion binding to protein is generally entropically driven, the overall enthalpy of our case reveals an exothermic event (Δ*H* < 0), indicating that Ca^2+^ binding to testicans is probably coupled to an enthalpically favorable formation of strong electrostatic bonds, leading to a more compact protein structure. The EF2-hand motif of all testicans appears to be incapable of calcium binding due to deviances from the consensus motif at sites, critical for calcium ion coordination, particularly at the Y position which is, in testicans, occupied by Phe (testican-1) or Tyr residue (testican-2 and -3). A similar analysis performed on testican-1 already provided clues for this [40], however, here we extended it to all three family members and provided a structural explanation. In the model of EF2 of testican-2, the presence of Tyr at position Y prevents calcium coordination via the side-chain distal oxygen atom and, at the same time, affects the conformation of the Asp residue at X position, again impairing its ability to participate in calcium coordination sphere.

By employing disorder prediction (all three testicans) and small-angle X-ray-scattering measurements (T2ΔC), we demonstrated that the N- and C-terminal regions, unique for testicans, are most likely structurally disordered. These regions may become structurally ordered upon, for example, binding to other ECM proteins, however, for the C-terminal region with a highly negative net charge, this is unlikely. On the other hand, the domain triplet FS–EC–TY with the calcium-binding site within the centrally located EC adopts a more compact structure which is additionally stabilized upon calcium binding, as revealed by decreased *R*_g_ and D_max_ parameters as well as characteristics of the disorder-indicating Kratky plot based on the SAXS data. Since extracellular Ca^2+^ concentrations in humans are in the range of 1.1–1.4 mM [43], which is much higher than the *K*_d_ value of testican-2 for calcium (17 µM), it is clear that calcium binding to testican-2 has solely a structure stabilizing role and is not a part of a putative regulation system involving the binding/release of calcium ions to/from testican-2. We conclude that, within ECM, testicans are always in a calcium-loaded form, and that calcium binding has a purely structure-stabilizing role.

Using SAXS data and homology models of individual FS, EC and TY domains, we constructed an integrative model of T2ΔC. In this model, represented by an ensemble of four models accounting for the inherent flexibility of the protein studied, the FS, EC and TY domains form a moderately compact core with a high probability of interdomain interactions. The EOM-generated models exhibit a protrusion accounting for the N-terminal region which, according to predictions and SAXS data, does not have a stable structure. This is also indicated by the P(r) function, which exhibits a tail at high values of radius, and therefore deviates from the curve characteristic for globular proteins where the tail is not as pronounced [44,45].

Due to the account of the previously demonstrated cell-behavior-modulating action of some SPARC family members, we addressed the possibility of such a modulation activity in testicans. For example, SPARC-treated non-small-cell lung cancer cells CL1-5 and H1299 migrate more rapidly [46], and hevin, the closest SPARC family member, has exactly the opposite mode of action, since migration is enhanced in hevin-null primary dermal fibroblasts [47]. These contradicting activities are intriguing, and since research done by other groups implicated full-length testican-1 promotes cell migration in vitro [5], we addressed this action in both testican-1 and -2. Cell exclusion zone assay revealed that both investigated testican paralogues (testican-1 and testican-2) indeed enhance cell migration. Since the truncated forms of testican lacking the N- and C-terminal regions had the same effect as the full-length form, the cell-migration-enhancing functionality can be attributed to the central FS–EC–TY domain triplet. Apart from testicans, there are several other proteins with unstructured regions that affect important cellular functions together with migration, including the majority of proteins that are important in cancer development, such as the members of CIP/KIP family [48,49]. Our results are in line with previous studies, which demonstrated that it is the protein part of testican-2 that mediates cellular functions like neurite outgrowth and not the attached GAG chains [20]. A similar functionality has been demonstrated for the BM-40/SPARC which contains the FS–EC domain pair, however it lacks the TY domain [46,50]. It is likely that also in testicans this functionality is linked to the FS–EC domain pair and not to the TY region. Interestingly, experiments on testican-3 using cell cultures showed that while the testican protein core is critical for ECM-testican interactions the GAG chains inhibit these interactions. Therefore, it seems that there is an intricate interplay between testican core protein, attached GAGs and the components of ECM which in turn may affect the aforementioned testican functionality.

Combined, our results indicate that testicans have a calcium-stabilized moderately compact core represented by the FS–EC–TY domain triplet. In isolated protein, both N- and C-terminal regions are disordered. The FS–EC–TY domain core domain triplet is also accountable for the cell-migration-promoting effect of testicans. The results offer a novel structural insight into testicans and, at the same time, provide an excellent basis for future research at the structure–function level of all three family members.

## 4. Materials and Methods

### 4.1. Cloning, Expression and Purification

cDNA of human testican-1 (UniProt ID Q08629-1.1) was kindly provided by C. J. S. Edgell (University of Carolina, USA); cDNA for human testican-2 (UniProt ID Q92563-1.1) was obtained from KAZUSA DNA Research Institute, Japan (clone ID KIAA0275) [51]. The cDNAs were used as a template to prepare one testican-1 construct (T1ΔC, residues 22–379) and three testican-2 constructs: T2 (residues 23–424), T2ΔC (residues 23–379), and T2ΔNC (residues 84–379) (Table S1). All of them had an N-terminal fusion composed of honeybee melittin signal peptide (replacing the native signal peptide), His_6_-tag and a TEV protease cleavage site (sequence STENLYFQ plus linker GAS). Recombinant bacmids were generated by transposition (donor plasmid pFastBac1) in *E. coli* DH10MultiBac cells [52]. The bacmids were, in turn, used to prepare recombinant baculoviruses by transfection into *Spodoptera frugiperda* cell line Sf9 using the TurboFect transfection reagent (Thermo Fisher Scientific, Vilnius, Lithuania).

For protein expression, Sf9 cells were grown in suspension cultures in serum-free Insect-XPRESS medium (Lonza, Verviers, Belgium) and infected with amplified recombinant baculovirus at a cell density of 1.8 × 10^6^ cell/mL and multiplicity of infection of 5. The medium containing the secreted recombinant proteins was harvested after 72 h using centrifugation (10 min at 6000× *g*). The pH of the medium was adjusted to 8.0 by the addition of 2 M Tris/HCl pH 8.0, and phenylmethylsulfonyl fluoride (PMSF) was added to a final concentration of 0.1 mM. Precipitated medium components were removed using centrifugation at 10,000× *g*, 20 min, and the supernatant was loaded onto 5 mL prepacked cOmplete His-Tag Purification Column (Roche, Switzerland). The column was washed with 50 mM Tris, 150 mM NaCl, pH 7.4, and bound proteins were eluted by gradually raising imidazole concentration to 500 mM. His_6_-tag was cleaved off using His_6_-tagged TEV protease mutant S2256N (mass ratio TEV protease:substrate was 1:50) during an overnight dialysis at 4 °C against buffer 50 mM Tris, 150 mM NaCl, 3 mM EDTA, 2 mM 2-mercaptoethanol, pH 7.6. The His_6_-tag-free proteins were recovered as a flow-through after applying the cleavage mixture onto 1 mL prepacked cOmplete His-Tag Purification Column (Roche, Switzerland). As a final purification step, size exclusion chromatography was performed using Superdex 200 Increase 10/300 column (GE Healthcare Bio-Sciences, Uppsala, Sweden), equilibrated in 20 mM HEPES, 150 mM NaCl, pH 7.4, with or without 3 mM CaCl_2_ (Figure S2). Protein samples were aliquoted and stored at −80 °C. The apparent molecular weight determined using SDS-PAGE (Figure S2, inset) corresponds to the one calculated from amino acid sequence, which indicates the absence of extensive glycosylation even for the GAG attachment sites containing T2 construct, as already reported earlier [39].

### 4.2. Calcium-Binding Analysis

Calcium-binding affinity and thermodynamic parameters were determined using isothermal titration calorimetry (ITC). For measurements, C-terminally truncated testican-2 was used (T2ΔC). Prior to measurements, protein sample was extensively dialysed against ITC buffer (20 mM HEPES pH 7.4, 150 mM NaCl) in the presence of Chelex beads (Sigma-Aldrich, St. Lous, MO, USA) to ensure complete removal of calcium ions. For preparation of the titrant solution, completely dry CaCl_2_ (drying at 700 °C) was dissolved in the ITC buffer.

ITC experiments were performed using NanoITC system (TA Instruments, New Castle, DE, USA). The sample cell was loaded with 26.9 µM protein solution and syringe was filled with 2.5 mM CaCl_2_ solution. A total of 6 µL of titrant was injected into the reaction cell at 6 min intervals, 37 times under constant stirring at 20 °C. The background was measured by averaging the heat of the last five injections at the end of the titration experiment, which corresponds to the heat of dilution, and subtracted from the individual measurements to obtain the effective heat of binding. The thermodynamic parameters ΔH, ΔS and ΔG were calculated according to a single-site model included in the NanoAnalyze software (TA Instruments, New Castle, DE, USA), and the data were visualized using OriginPro (OriginLab, Northampton, MA, USA).

Sequence alignment of EF-hand motifs of testicans and BM-40 was prepared using Clustal Omega [53]. The initial structural models of EF-hands of testican-2 were prepared using I-TASSER server [54] using BM-40 structure [10] as a template, and then manually refined using UCSF Chimera [55] to accommodate a Ca^2+^ ion.

### 4.3. Sequence Analysis and Disorder Prediction

Compositional bias of the testican-2 sequence was assessed using program fLPS [32]. Reference compositions were obtained by a composition analysis of several sets of sequences—vertebrate and human proteins in UniProt/SwissProt, both with and without annotation of localization in extracellular matrix). Using all four generated reference sets, the same region with highest compositional bias was detected—residues 316–416.

For disorder prediction, three different servers were used: IUPred2A [35], DisEMBL 1.5 [36], and PrDOS [37]. In all three cases, the sequence of mature testican form was used as an input (residues 23-424, UniProt ID Q92563-1.1).

### 4.4. Small Angle X-ray Scattering and Modeling

SAXS data were collected at Petra III, DESY, beamline P12 (Hamburg, Germany). Scattering was measured for T2ΔC construct in the presence and absence of calcium ions and at protein concentrations ranging from 0.6 to 10.6 mg/mL. Calcium-free samples were prepared in the same manner as for the ITC experiments. Matching reference buffer was prepared by three subsequent dialysis steps, each overnight against an SAXS buffer (20 mM HEPES, pH 7.4, 150 mM NaCl, 5% (*v*/*v*) glycerol, and, optionally, 3 mM CaCl_2_). As a reference, scattering of the bovine serum albumin (BSA) solution was measured (3.64 mg/mL). Scattering profiles are shown in Figure S4. Data collection parameters are summarized in Table S1. Data were also collected for two other testican-2 forms—T2 (full length) and T2ΔNC—however, they exhibited aggregation, making data analysis impossible.

For data analysis, the ATSAS 3.0.2 software package was used [56]. First, experimental curves obtained at different concentrations were used to determine the radius of gyration (*R*_g_) (Figure S6) and forward scattering (*I*_0_). An increase in the radius of gyration (*R*_g_) as well as in molecular weight (M_w_) calculated from forward scattering at higher concentrations indicates a minor concentration effect (Figure S5), that was suppressed with extrapolation to infinite dilution, which minimized the effect of inter-particle attraction or aggregation and preserved a good signal-to-noise ratio. Next, pair distance distribution function (P(r)) and the corresponding maximum particle size parameter (D_max_) were determined using the GNOM software [57]. Theoretical molecular envelopes were reconstructed by ab initio modeling using the program DAMMIF [58] running for 20 cycles, where scattering from the calculated models was fitted against the experimental scattering. The most typical model was selected by pairwise comparison of the normalized spatial discrepancy (NSD), and averaged by DAMAVER software [59]. More detailed models comprised of the individual domains were prepared using the EOM program [60] on the basis of the processed scattering data, homology models of the individual domains (described below), and sequence data (residues 23–379). Molecular envelopes and structural models were visualized using UCSF Chimera [55].

The homology models of FS (residues 99–182), EC (residues 199–307) and TY domain (residues 313–379) of testican-2 were prepared using I-TASSER modeling server [54] with automatic template structure selection.

SAXS data and models were deposited to the SASBDB [61] under the accession codes SASDKJ2 (Ca^2+^-free T2ΔC) and SASDKK2 (Ca^2+^-loaded T2ΔC).

### 4.5. Cell Exclusion Zone Assay

For cell assays, the glioma cell line U373 was used. Cells were maintained in Dulbecco’s Modified Eagle’s Medium (DMEM; Gibco, Thermo Fisher Scientific, Life Technologies, Paisley, UK) supplemented with 10% fetal bovine serum (Sigma-Aldrich, Steinheim, Germany) and 1% Pen-strep (Gibco, Thermo Fisher Scientific, Life Technologies, Grand Island, NY, USA) at 37 °C in a humidified incubator with 5% CO_2_. 

Cell exclusion zone assay was performed using 24-well plates with 2-well culture silicone inserts (ibidi, Gräfelfing, Germany). Here, each insert consists of two wells that are separated with a 500-µm-thick wall. Both wells were inoculated with U373 cells pretreated with 10 µg/mL mitomycin c (Sigma-Aldrich, St. Louis, MO, USA) to block cell proliferation. After reaching 80% confluence, the inserts were removed, thereby creating a 500-µm cell-free gap. Cell debris was removed by washing with serum-free medium twice. The cells were then cultured in serum-free medium (170 µL per well) supplemented with 10 µg of a sterile-filtered solution of testican construct per well (T1ΔC, T2ΔC, T2ΔNC). BSA was used as a negative control. Cell migration was monitored using TCS SP8 confocal microscope (Leica Microsystems, Wetzlar, Germany) at 12, 24 and 40 h timepoints. The obtained microscopic images were analyzed using ImageJ MRI Wound Healing Tool [62]. Average gap closure was expressed as percentage closure relative to original wound size and was calculated using the following equation

$$\left(1-\frac{a}{b}\right)\times 100$$

where *b* and *a* are cell-free areas before and after closure, respectively [63]. Results are expressed as means ± SD. One-way ANOVA followed by Tukey’s post hoc tests was used for statistical evaluation.

## Figures and Tables

**Table 1:** Thermodynamic parameters for calcium binding to C-terminally truncated testican-2 (T2ΔC). Δ*H*, Δ*S* and Δ*G* correspond to changes in enthalpy, entropy and Gibbs free energy, respectively, and *T* denotes temperature.

*K*_d_ [µM]	n	Δ*H* [kcal/mol]	*T*Δ*S* [kcal/mol]	Δ*G* [kcal/mol]
17.1 ± 3.4	1.3 ± 0.2	−17.64 ± 2.4	11.25 ± 0.22	−6.39

## Supplementary Materials

Supplementary materials can be found at https://www.mdpi.com/1422-0067/21/24/9413/s1.

## Abbreviations

BSA	bovine serum albumin
D_max_	maximum dimension
EC	extracellular calcium-binding (domain)
ECM	extracellular matrix
EOM	ensemble optimization method
FS	follistatin (domain)
*G*	Gibbs free energy
GAG	glycosaminoglycan
*H*	enthaply
*I*; *I*_0_	scattered intensity; extrapolated scattering intensity at *q* = 0
ITC	isothermal titration calorimetry
*K*_d_	dissociation constant
MT-MMP	membrane-type matrix metaloprotease
M_w_	molecular weight, in Da (daltons)
PDDF, P(r)	pair distance distribution function
*q*	momentum of transfer
*R*_g_	radius of gyration
*S*	entropy
SAXS	small angle X-ray scattering
SEC	size-exclusion chromatography
*T*	temperature
TEV	tobbaco etch virus
TY	thyroglobulin type-1 (domain)
