# Supplementary material for: The Central Region of Testican-2 Forms a Compact Core and Promotes Cell Migration
Source: Int J Mol Sci. 2020 Dec 10;21(24):9413. doi: 10.3390/ijms21249413 (PMC7763218; doi:10.3390/ijms21249413)
Supplement: Supplementary file 1 [file ijms-21-09413-s001.pdf]

SUPPLEMENTARY MATERIALS

Compact testican core enveloped with flexible terminal regions is promoting cell migration

Anja Krajnc [1], Aljaž Gaber [1], Brigita Lenarčič [1,2] and Miha Pavšič [1,*]

[1] Department of Chemistry and Biochemistry, Faculty of Chemistry and Chemical Technology, University of Ljubljana, 1000 Ljubljana, Slovenia; anja.krajnc@fkkt.uni-lj.si (A.K.), brigita.lenarcic@fkkt.uni-lj.si (B.L.), miha.pavsic@fkkt.uni-lj.si (M.P.)

[2] Department of Biochemistry, Molecular and Structural Biology, Institute Jožef Stefan, 1000 Ljubljana, Slovenia

* Correspondence: miha.pavsic@fkkt.uni-lj.si; (M.P.)

Supplementary Materials and Methods

Sequence alignment of testicans

```
T2  -EGDAKGLKEGETPGNFMEDEQWLSSISQYSGKIKHWNRF  61
T1  RHLDALAGGAGPNHGNFLDNDQWLSTVSQYDRDK-YWNRF  60
T3  AAAAVAAAGGRSDGGNFLDDKQWLTTISQYDKEVGQWNKF  61
        . .       ***:::.***:::***. .   **:*

                                1    2     1
T2  RDEVEDDYIKSWEDNQQGDEALDTTKDPCQKVKCSRHKVC  101
T1  RD---DDYFRNWNPNKPFDQALDPSKDPCLKVKCSPHKVC  97
T3  RDEVEDDYFRTWSPGKPFDQALDPAKDPCLKMKCSRHKVC  101
    **   ***::.*. .:  *:*** :**** *:*** ****

             2                            1
T2  IAQGYQRAMCISRKKLEHRIKQPTVKLHGNKDS----ICK  137
T1  VTQDYQTALCVSRKHLLPRQKKGNVAQKHWVGPSNLVKCK  137
T3  IAQDSQTAVCISHRRLTHRMKEAGVDHRQWRGP-ILSTCK  140
    ::*. * *:*:*:::*  * *:  *  :          **

     2        3          2      1
T2  PCHMAQLASVCGSDGHTYSSVCKLEQQACLSSKQLAVRCE  187
T1  PCPVAQSAMVCGSDGHSYTSKCKLEFHACSTGKSLATLCD  177
T3  QCPVVYPSPVCGSDGHTYSFQCKLEYQACVLGKQISVKCE  180
     * :.  : *******:*:  **** :**  .*.::. *:

      3
T2  GPCPCPTEQAATSTADGKPETCTGQDLADLGDRLRDWFQL  217
T1  GPCPCLPEPEPPKHKAERS-ACTDKELRNLASRLKDWFGA  216
T3  GHCPCPSDKPTSTSRNVKR-ACSDLEFREVANRLRDWFKA  219
    * ***  :            :*:. :: ::..**:***

T2  LHENSKQNGSASSVAGPASG-LDKSLGASCKDSIGWMFSK  256
T1  LHEDANRVIKP-TSSNTAQGRFDTSILPICKDSLGWMFNK  255
T3  LHESGSQNKKTKTLLRPERSRFDTSILPICKDSLGWMFNR  259
    ***...:  .  :      . :*.*:   ****:****.:

T2  LDTSADLFLDQTELAAINLDKYEVCIRPFFNSCDTYKDGR  296
T1  LDMNYDLLLDPSEINAIYLDKYEPCIKPLFNSCDSFKDGK  295
T3  LDTNYDLLLDQSELRSIYLDKNEQCTKAFFNSCDTYKDSL  299
    ** .**:** :*: :* *** * * : :*****::**.

                     1
T2  VSTAEWCFCFWREK-PPCLAELERIQIQEAAKKKPGIFIP  335
T1  LSNNEWCYCFQKPGGLPCQNEMNRIQKLSKGKSLLGAFIP  335
T3  ISNNEWCYCFQRQQDPPCQTELSNIQKRQGVKKLLGQYIP  339
    :*. ***:** :    **  *:..**  .  *.  * :**

     1         2      2 3
T2  SCDEDGYYRKMQCDQSSGDCWCVDQLGLELTGTRTHGSPD  375
T1  RCNEEGYYKATQCHGSTGQCWCVDKYGNELAGSRKQGAVS  375
T3  LCDEDGYYKPTQCHGSVGQCWCVDRYGNEVMGSRINGVAD  379
     *:*:***:  **. * *:*****: * *: *:* :*  .

    3
T2  CDDIVGFSGDFGSG--VGWEDEEEKETEEAGEEAE-----  408
T1  CEEEQETSGDFGSGGSVVLLDDLEYERELGPKDKEGKLRV  415
T3  CAIDFEISGDFASGDFHEWTDDEDDEDDIMNDEDE----   414
    *      ****.**      *: : * :    : *

T2  --------EEEGEAGEADDGGYIW                  424
T1  HTRAVTEDDEDEDDDKEDEVGYIW                  439
T3  -IEDDDEDEGDDDDGGDDHDVYI-                  436
            : : : .  *.  **
```

Figure S1. Sequence alignment of testican-2 (T2), testican-1 (T1) and testican-3 (T3). The three central domains are shown in green (follistatin, FS), orange (calcium-binding, EC) and blue (thyroglobulin, TY), and glycosylation sites—as annotated in UniProt—denoted by squares. Cysteine residues are shown in yellow and numbered (each region/domain with its of numbering, color-coded), the same two numbers within a region/domain denote cysteine residues forming a disulfide bond. Alignment was prepared using Clustal Omega. Domains were predicted with InterProScan.

Size-exclusion chromatography and PAGE of purified constructs

Recombinant proteins used for all described experiments were purified using immobilized metal affinity chromatography and size exclusion chromatography, and the purity was analyzed using SDS-PAGE.

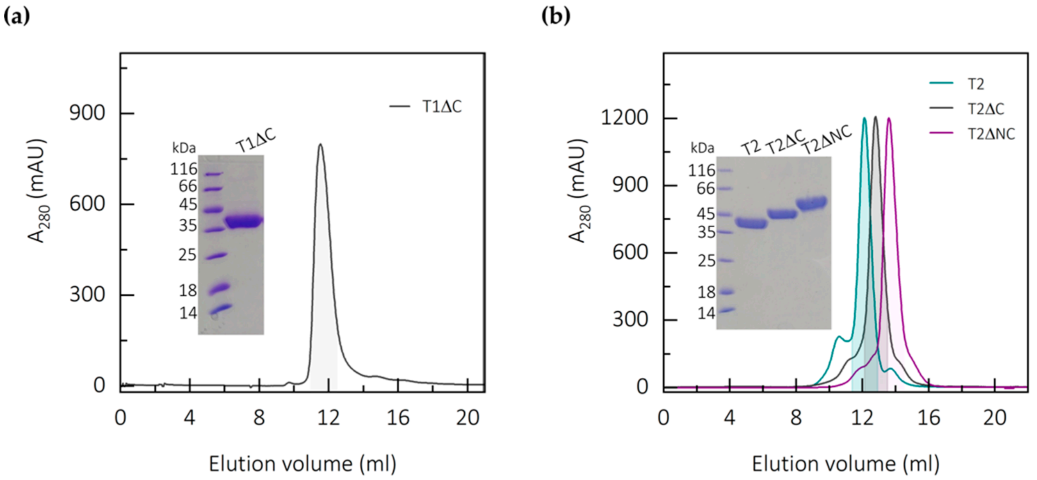

Figure S2. Sample purity analysis of **(a)** testican-1 and **(b)** testican-2 constructs. Size-exclusion chromatography and SDS-PAGE analysis of the pooled fractions indicate high purity of all of the samples. Size-exclusion chromatography was performed using SEC 200 Increase 10/300 column (GE Healthcare). SDS-PAGE analysis was carried out in the presence of 2-mercaptoethanol.

Disorder tendency plots for testican-1, -2 and -3

Disorder tendency prediction conducted for testican-1 and -3 revealed their N- and C-terminal regions are disordered, similarly as in testican-2.

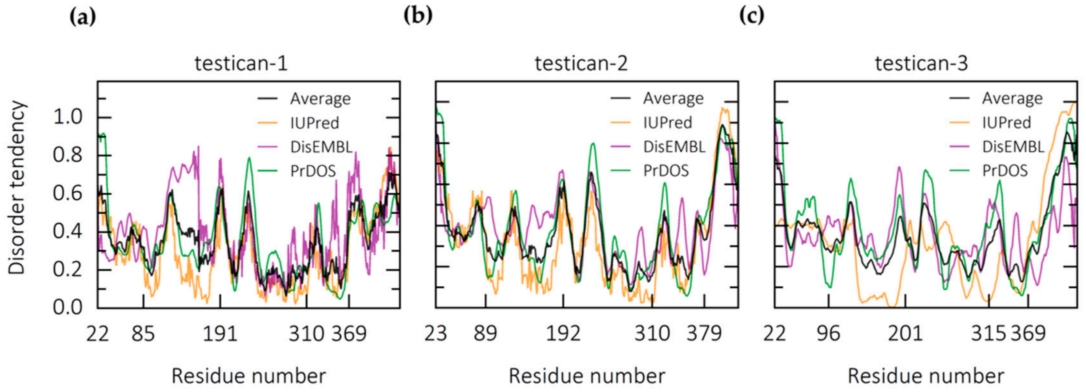

Figure S3. Prediction of disordered regions. **(a)** Disorder tendency plot for testican-1, **(b)** testican-2, and **(c)** testican-3. Profile comparison reveals a similar pattern of disordered regions. The disorder tendency plot was calculated using various software. The average value of disorder tendency is represented with a black line. Marked residue numbers represent domain border area and correspond roughly to increased disorder tendency.

SAXS data analysis

For each protein sample a dataset concentration series were collected in order to evaluate monodispersity. Due to observed moderate concentration effects, data was processed by extrapolation to infinite dilution.

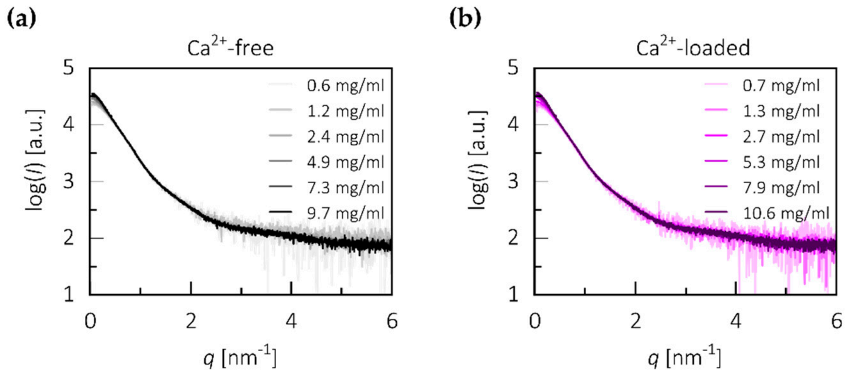

Figure S4. Scattering profiles (logarithmic scattering I vs. momentum transfer q) at different concentrations of T2ΔC after background subtraction and concentration normalization. (**a**) Scattering profiles in the absence of Ca^{2+} ions. (**b**) Scattering profiles in the presence of Ca^{2+} ions. Moderate decrease in intensity at low q-values with increasing protein concentration indicates some concentration effects are present at higher protein concentrations.

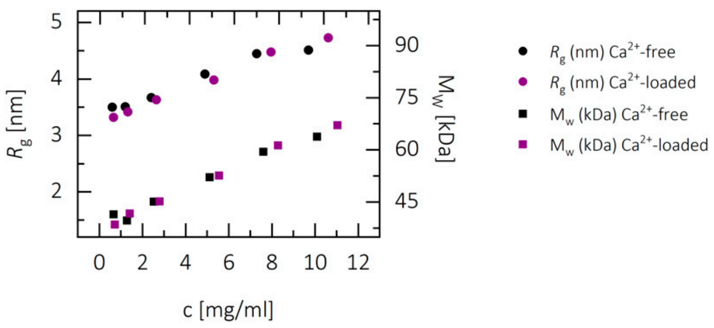

Figure S5. The concentration effect observed as increasing R_g (circles) and M_w (squares; values calculated using DATMOV) with increasing concentration of the protein for Ca^{2+}-free (black) and Ca^{2+}-loaded T2ΔC (purple).

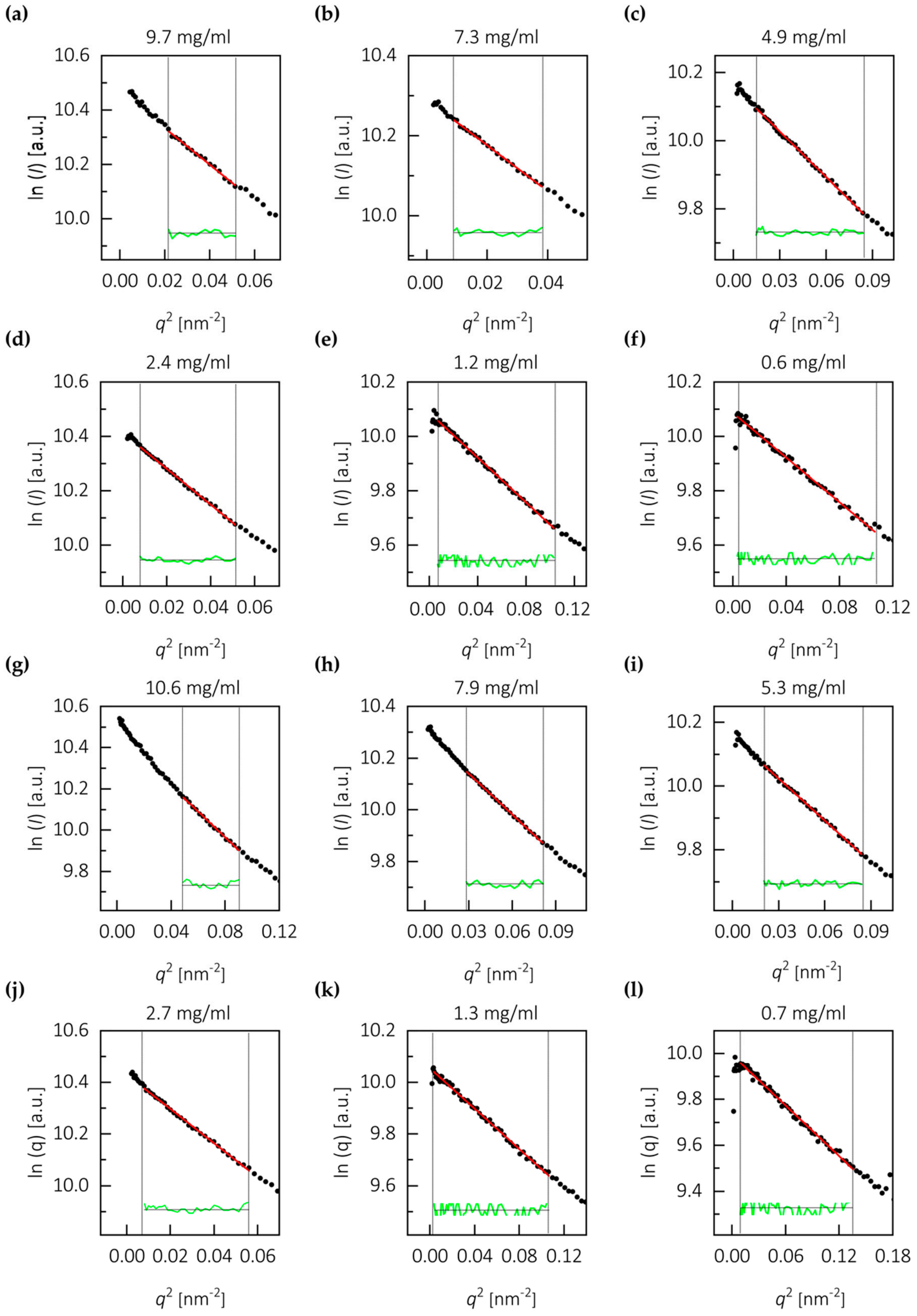

Figure S6. Guinier analysis. Guinier plots show linearity in the low q-region. Experimental data are shown as black dots, the fit of the linear region in red, and the residuals in green. (**a-f**) Ca^{2+}-free T2ΔC. (**g-l**) Ca^{2+}-loaded T2ΔC. Protein concentration is indicated above each plot.

SAXS data collection, structural parameters and modeling

Table 1. SAXS data collection, analysis and derived structural parameters.

**Data collection parameters**		
Beamline	Petra III, DESY (Hamburg, Germany)	
Beamline	EMBL P12	
Detector	Pilatus 2M	
Beam size [mm]	0.12 × 0.2	
Wavelength [nm]	0.124	
Sample to detector distance [m]	1.6	
Momentum of transfer q range [nm^{-1}]	0.0048–9.7 nm^{-1}	
Exposure time per frame [s]	0.045 (120 frames)	
Temperature [°C]	20	
Mode	Standard batch mode	
**Overall structural parameters**		
Sample	**T2ΔC Ca^{2+}-free**	**T2ΔC Ca^{2+}-loaded**
Concentration range	0.6–9.7 mg/ml<br>(0.015–0.24 mM)	0.7–10.6 mg/ml<br>(0.016–0.27 mM)
Buffer	20 mM Na-HEPES, 130 mM NaCl, 5 % (v/v) glycerol, pH 7.4	20 mM Na-HEPES, 130 mM NaCl, 3 mM $CaCl_2$, 5 % (v/v) glycerol, pH 7.4
R_g (Guinier) [nm]	3.41 (*zc*)<br>3.5 (0.015 mM)<br>4.09 (0.12 mM)<br>4.51 (0.24 mM)	3.24 (*zc*)<br>3.32 (0.016 mM)<br>3.98 (0.13 mM)<br>4.73 (0.27 mM)
R_g (PDDF) [nm]	3.49 (*zc*)<br>3.63 (0.015 mM)<br>4.32 (0.12 mM)<br>4.98 (0.24 mM)	3.35 (*zc*)<br>3.44 (0.016 mM)<br>4.28 (0.13 mM)<br>5.14 (0.27 mM)
D_{max} [nm]	14 (*zc*)<br>14 (0.015 mM)<br>17 (0.12 mM)<br>20.5 (0.24 mM)	12.6 (*zc*)<br>12.7 (0.016 mM)<br>16 (0.13 mM)<br>21 (0.27 mM)
M_w (DATMOV) [kDa]	41.9 (*zc*)<br>41.4 (0.015 mM)<br>51.1 (0.12 mM)<br>63.8 (0.24 mM)	40.1 (*zc*)<br>38.5 (0.016 mM)<br>52.6 (0.13 mM)<br>67.1 (0.27 mM)
M_w (Porod) [kDa]	46.6 (*zc*)<br>47.4 (0.015 mM)<br>63.1 (0.12 mM)<br>80.1 (0.24 mM)	44.1 (*zc*)<br>44.2 (0.016 mM)<br>62.5 (0.13 mM)<br>86.2 (0.27 mM)
M_w (sequence, monomer) [kDa]	40.0	

(continued)

$q = 4\pi \sin(\theta/\lambda)$, where 2θ is the scattering angle and λ is the wavelength. For both samples derived parameters are listed for lowest, highest and one middle concentration point, plus for the data extrapolated to zero concentration (*zc*).

(continued)

Sample	**T2ΔC Ca^{2+}-free**	**T2ΔC Ca^{2+}-loaded**
**Software employed**		
Primary data reduction	SaxsAnalysis pipeline system	
Data processing and modeling	ATSAS 3.0.2	
Processing	PRIMUS, GNOM 5.0	
Ab initio modeling	DAMMIF, DAMMIN 5.3	
Ensemble modeling	EOM (RANCH 2.1, GAJOE 2.1)	
R_g (EOM ensemble) [nm]		3.33
D_{max} (EOM ensemble) [nm]		12.7
SASBDB accession code	SASDKJ2	SASDKK2

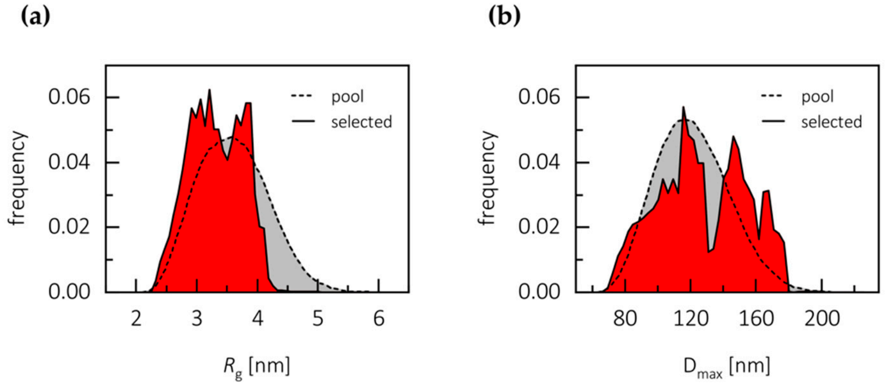

Figure S7. (**a**) R_g and (**b**) D_{max} distribution of models selected from the pool generated during EOM modeling.

Cell exclusion zone assay

Microscopic images were analyzed using an optimized ImageJ plugin – Wound Healing Size Tool by Suarez-Arnedo et.al., which enabled quantification of the cell-free surface area. For each condition the assay was performed in triplicate to ensure reproducibility and permit statistical evaluation of the results.

Table 2. Measurements of the cell-free gap.

**Cell free surface in μm²**						
Added constructs	Time [h]	1. repeat	2. repeat	3. repeat	Mean	SD
BSA	0	86495	85014	82588	84699	1610
	12	89886	83564	86841	86793	2581
	24	54826	70100	63444	62790	6252
	40	32896	21243	32237	28792	5344
T1ΔC	0	87500	84124	85883	85835	1378
	12	64516	69801	61976	65431	3259
	24	5370	3842	4261	4491	644
	40	3198	2035	778	2003	988
T2	0	82905	88417	85001	85441	2271
	12	79665	85454	83883	83000	2444
	24	9870	15637	9013	11506	2941
	40	2945	3198	678	2273	1133
T2ΔC	0	85427	82564	87152	85047	1892
	12	73799	80459	82624	78960	3755
	24	5631	20510	25891	17344	8568
	40	2245	3200	3198	2881	449
T2ΔNC	0	90117	81778	87770	86555	3511
	12	74476	74953	60141	69856	6872
	24	14050	6264	18264	12859	4970
	40	9949	1699	1996	4548	3821
T1ΔC + T2	0	84251	85692	80222	83389	2313
	12	81655	76856	83691	80734	2865
	24	14569	9632	16221	13474	2799
	40	2951	8963	9985	7299	3103
T1ΔC + T2ΔC	0	80256	85123	90254	85211	4082
	12	68546	64236	77569	70117	5555
	24	15569	7256	8569	10464	3648
	40	2951	8963	9985	7299	3103
T1ΔC + T2ΔNC	0	90117	8178	87770	86555	3511
	12	74476	74953	60141	69856	6872
	24	14050	6264	18264	12859	4970
	40	9949	1699	1996	4548	3821

Table 3. Average gap closure.

Average gap closure [%]			
	Time [h]		
**Added constructs**	12	24	40
Neg. control	-	25.9	66
T1ΔC	23.8	94.8	97.7
T2	2.9	86.5	97.3
T2ΔC	7.1	79.6	96.6
T2ΔNC	19.3	85.1	94.7
T1ΔC + T2	3.2	83.3	91.2
T1ΔC + T2ΔC	17.7	87.7	92.9
T1ΔC + T2ΔNC	10.2	85.1	94.2

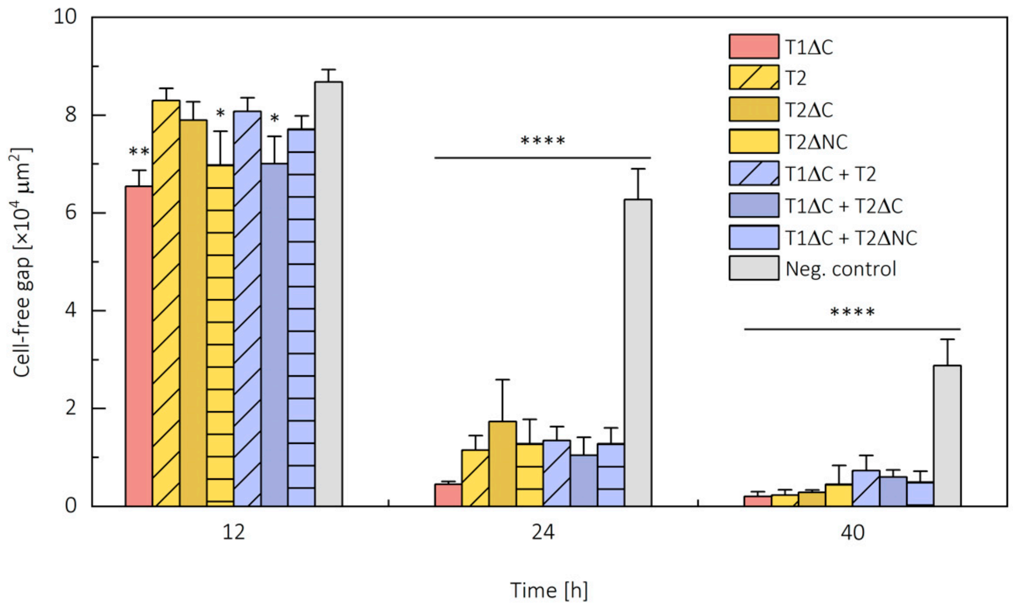

Figure S8. Quantified cell-free area in the presence of different testican constructs either separately or in various combinations at different time points, indicating there is no significant difference in cell migration between conditions with single testican construct and conditions with two-construct combinations. Values represent mean values of 3 independent measurements with s.d., *$p < 0.0332$, **$p < 0.0021$, ****$p < 0.0001$, one-way ANOVA test with Tukey post hoc analysis.
